# A novel GCaMP6f-RCS rat model for studying electrical stimulation in the degenerated retina

**DOI:** 10.3389/fcell.2024.1386141

**Published:** 2024-04-22

**Authors:** Tamar Azrad Leibovitch, Nairouz Farah, Amos Markus, Yossi Mandel

**Affiliations:** ^1^ Bar Ilan Institute for Nanotechnology and Advanced Materials (BINA), Bar Ilan University, Ramat Gan, Israel; ^2^ Faculty of Life Sciences, School of Optometry and Visual Science, Bar Ilan University, Ramat Gan, Israel

**Keywords:** retinal protheses, calcium imaging, electrical stimulation, retinal degeneration, GCaMP6f-RCS

## Abstract

**Background:** Retinal prostheses aim to restore vision by electrically stimulating the remaining viable retinal cells in Retinal Degeneration (RD) cases. Research in this field necessitates a comprehensive analysis of retinal ganglion cells’ (RGCs) responses to assess the obtained visual acuity and quality. Here we present a novel animal model which facilitates the optical recording of RGCs activity in an RD rat. This model can significantly enhance the functional evaluation of vision restoration treatments.

**Methods:** The development of the novel rat model is based on crossbreeding a retinal degenerated Royal College of Surgeons (RCS) rat with a transgenic line expressing the genetic calcium indicator GCaMP6f in the RGCs. Characterization of the model was achieved using Optical Coherence Tomography (OCT) imaging, histology, and electroretinography (ERG) at the ages of 4, 8, and 12 weeks. Additionally, optical recordings of RGCs function in response to *ex-vivo* subretinal electrical stimulations were performed.

**Results:** Histological investigations confirmed the high expression of GCaMP6f in the RGCs and minimal expression in the inner nuclear layer (INL). OCT imaging and histological studies revealed the expected gradual retinal degeneration, as evident by the decrease in retinal thickness with age and the formation of subretinal debris. This degeneration was further confirmed by ERG recordings, which demonstrated a significant decrease in the b-wave amplitude throughout the degeneration process, culminating in its absence at 12 weeks in the GCaMP6f-RCS rat. Importantly, the feasibility of investigating subretinal stimulation was demonstrated, revealing a consistent increase in activation threshold throughout degeneration. Furthermore, an increase in the diameter of the activated area with increasing currents was observed. The spatial spread of the activation area in the GCaMP6f-RCS rat was found to be smaller and exhibited faster activation dynamics compared with the GCaMP6f-LE strain.

**Conclusion:** This novel animal model offers an opportunity to deepen our understanding of prosthetically induced retinal responses, potentially leading to significant advancements in prosthetic interventions in visual impairments.

## Introduction

Outer retinal degenerative diseases are the leading cause of blindness in the Western world and are characterized by the degeneration of the photoreceptors (PRs) while largely sparing the inner retinal cells. The most common of these diseases are Age-related Macular Degeneration (AMD) (Flaxman et al., 2017) and a group of hereditary retinal dystrophy called Retinitis Pigmentosa (RP) (Flaxman et al., 2017).

Numerous vision restoration strategies are currently under pre- or clinical evaluation. A principal vision restoration strategy, cell replacement therapy, relies on replacing the diseased RPE or photoreceptor cells (or both) (Sharma and Jaganathan, 2021); others, such as optogenetics (Sahel et al., 2021; Parnami and Bhattacharyya, 2023) and optical switches (Tochitsky et al., 2018) are based on optically stimulating the remaining neurons. An alternative promising vision restoration strategy is the electrical stimulation of the remaining viable retinal circuitry using retinal prostheses composed of an electrode array implanted in the sub-, epi retinal, or suprachoroidal spaces (Palanker and Goetz, 2018; Palanker, 2023; Wu et al., 2023). The various stimulus parameters (Muralidharan et al.; Guo et al., 2018; Twyford et al., 2014) and electrode configurations (Mathieson et al., 2012; Boinagrov et al., 2014; Seo et al., 2023) determine the extent of the spatial spread of activation and whether stimulation induces direct activation of the RGCs or network activation; both are critical in determining the obtained resolution and quality of the restored vision. Therefore, investigating the induced RGCs responses is a common practice when studying retinal prosthetic and other vision restoration approaches.

To investigate the electrically induced responses of the RGCs, the need arises to simultaneously record the RGC responses using an additional multi-electrode array mounted on the RGCs side (Mathieson et al., 2012; Seo et al., 2023), which is technically challenging. Furthermore, the electrophysiological signals are contaminated with the stimulation artifacts, further complicating their analysis. In the epiretinal stimulation case, where the stimulating electrode is simultaneously used to acquire the RGCs activity, early onset activity is lost due to amplifier saturation and the stimulating artifact (Sim et al., 2014).

To overcome these challenges, efforts have shifted towards optically visualizing the RGCs activity by utilizing fluorescent reporters such as calcium indicators and voltage-sensitive dyes. Retrograde labeling of RGCs with the synthetic calcium indicator OGB has been demonstrated as a possible method for investigating the RGCs responses and for characterizing the spatial spread of the electrical stimulation by visualizing the fluorescence signal *ex vivo* (Behrend et al., 2009). This labeling approach, however, is time consuming and time limited by the expelling of the dye by the RGCs, thus suggesting the advantages of switching to genetically encoded calcium indicators (GECIs). More recently (Borghuis et al., 2011), the viral transfection of the RGCs with either GCaMP3 or similar indicators has been shown to provide a stable expression of the calcium indicators as well as valuable and precise information about the spatial patterns of the retinal activation (Borghuis et al., 2011; Weitz et al., 2013). In addition to these *in-vitro* investigations, the feasibility of imaging both the expression patterns and functionality of RGCs expressing GECI *in vivo* using adaptive (Bar-Noam et al., 2016; Li et al., 2022) optics as well as other systems (Schejter et al., 2012; Chang et al., 2016; Weitz et al., 2013; Cheong et al., 2018) has been demonstrated by several groups. This approach depends on the viral transfection of the GECIs, which limits the extent of the expression region and suffers from issues such as toxicity, non-uniform expression level, and decay over time (Dana et al., 2014; Li et al., 2022). Recent reports on transgenic mice (Dana et al., 2018; Blandford et al., 2019) introduced the exciting possibility of combining advancements in GECI to optically investigate RGCs activity. However, most of the work reported with both approaches has focused on mice, which have smaller eyes, compared with rats, making the transplantation of a retinal prosthesis challenging. Therefore, it is highly desirable to develop a transgenic rat by combining both a retinal degeneration model and a uniform and stable expression of the GECIs.

There are several available rodent models of retinal degeneration (RD) (Veleri et al., 2015; Di Pierdomenico et al., 2017). The RCS rat is a well-established RD model, which was described as a spontaneously inherited recessive mutation in the MERTK gene, encoding to tyrosine kinase receptor (Strauss et al., 1998; Wang et al., 2005; Ryals et al., 2017a; Di Pierdomenico et al., 2017; He et al., 2019; Nandrot et al., 2000; D’Cruz et al., 2000). This mutation manifests in defective phagocytosis of the photoreceptor’s outer segments by the RPE cells, which leads to a build-up of cellular debris in the subretinal space. This animal has been extensively studied; the gradual loss of electroretinogram signals throughout maturation has been widely reported, starting as early as postnatal day 21 (P21) and its complete loss by P90. This relatively slow degeneration (Ryals et al., 2017b) enables the development of a normal visual cortex, making the RCS rat an attractive animal model for both *in-vivo* and *ex-vivo* studies of vision restoration strategies, such as cell replacement (Salas et al., 2021), optogenetic (Tomita et al., 2007) and retinal prosthesis implantation (Lorach et al., 2014; Lorach et al., 2015; Arens-Arad et al., 2020).

Here we present the development of such an animal model based on the crossbreeding of two lines: the RCS rat (Gal et al., 2000) with a transgenic line of a GCaMP6f rat under the Thy1 promotor (Scott et al., 2018; Kaszas et al., 2021). We obtained an animal model with an RD expressing GCaMP6f in the RGCs. We report on the thorough characterization of this novel breed by imaging, histology, and electrophysiological studies. We further demonstrate the investigation of *ex-vivo* sub-retinal electrical stimulation at various times throughout the retinal degeneration process. We believe that the developed breed will prove to be a vital tool in the *ex-vivo* and *in-vivo* investigation of retinal stimulation strategies in animals with retinal degeneration.

## Methods

### Generating a GCaMP6f-RCS line

#### Animal model

This research involved the crossbreeding of two different rat strains. The first, namely, the RCS, is a spontaneously inherited retinal degeneration and a well-established model of pigment epithelium defect caused by a mutation in the MERTK Gene (Nandrot et al., 2000; D’Cruz et al., 2000). This strain was maintained by inbreeding. The second breed (Thy1-GCaMP6f) was purchased from the University of Missouri. It was generated in Janelia Research Campus by the injection of a linearized DNA containing the expression cassette into single-cell embryos before returning to host mothers, and is maintained by cross-breeding with Long-Evans rats (Scott et al., 2018).

Two breeding generations were required to establish the new GCaMP6f-RCS model, since the available transgenic GCaMP6f animal breed is hemizygous for GCaMP6f (possessing only one allele of the gene), and in the RCS model, only homozygous animals express an RD phenotype (the desired phenotype of the new model is GCaMP6f^+\−^RCS^-\-^). To obtain the GCaMP6f^+\−^RCS^-\-^ animal breed, a GCaMP6f^+\-^ male was crossbred with an RCS (MERTK^−\-^) female rat. After maturation and genotyping, two GCaMP6f^+\−^RCS^+\-^ males from the offspring of the first breeding were crossbred with another two RCS^−\-^ female rats. At this generation, 25% are GCaMP6f^+\−^RCS^-\-^, which is our desired breed. To maintain the desired colony, the GCaMP6f^+\−^RCS^-\-^ rats were crossbred with an RCS^−\-^ rat, yielding a colony with 50% GCaMP6f^+\−^RCS^-\-^ offspring.

### Genotyping–PCR DNA sampling and DNA extraction

At the age of 4–12 weeks, pups were lightly anesthetized with 3.5% isoflurane, and an ear sample was collected for DNA analysis (using a puncture and kept at −18°C until analysis). The samples were incubated at 95°C for 15 min in a solution containing 10 mL EDTA 0.5M pH = 8, 500 mL NaOH 1M, and 50 mL water. Afterwards, 100 mL of 40 mM Tris HCL at pH = 5 was added, and the samples were spun down.

#### Polymerase chain reaction (PCR)

Samples were prepared for a PCR reaction with 1 mL from the DNA sample prepared above, 1 mL dNTPs, 1 mL of primer pairs (Table 1 in the Supplementary Material), 10 mL DNA Polymerase (PCRBIO HS Taq DNA mix red, PCRBIOSYSTEM, #PB10.23–02) and 8 μL DDW. The PCR protocol was then optimized separately for RCS primers and for GCaMP6f primers according to the reactions described in Table 2 in the Supplementary Material.

#### Gel electrophoresis

First, 1% gel agarose (Hydragene, #R9012LE) was dissolved in TAEX1 (dissolved with DDW from TAEX50 (Biolab, #2050237500)) and then carefully heated in a microwave. When the solution was completely clear, 8 μL of GelGreen nucleic acid stain (Biotium, #41005) was added. The solution was poured into a mold and the gel comb was immediately inserted. The gel was cooled to room temperature for 20 min and then gently placed in the TBEX1 buffer-filled box. All samples were loaded in their wells including DNA markers, and an electrical current was applied via the power supply to the rear. After about 30 min, the gel was removed for imaging under ultraviolet light.

### Histological characterization

The eyes of GCaMP6f or GCaMP6f-RCS rats were enucleated and a small puncture, using a 27G needle, was made in the cornea, after which the eyes were incubated in 4% PFA for 24 h at 4°C or 1 h at room temperature. Next, the eyes were rinsed with PBS, followed by removing the anterior segment, yielding a posterior segment ready to be stained as described next.

For whole-mount staining, the posterior segment was prepared for immunochemistry by washing twice with PBS and then with PBS-TWEEN 0.5% (Sigma-Aldrich, St. Louis, MO) for 20 min. After overnight incubation in 0.01 g/mL Bovine albumin (LOT M8665, MP Biomedicals™) and 5% horse serum (#04-124-1A, Biological Industries) blocker solution, slides were incubated for three nights at 4°C with the primary antibodies (see Table 3 Supplementary Material). Samples were washed with PBS, followed by an overnight incubation with a secondary antibody. Next, samples were incubated in Hoechst 1:500 (BISBENZIMIDE H 33342, #14533-100MG, Sigma-Aldrich) for 30 min and then washed 3 times with PBS and finally gently flattened. The prepared sample was then mounted onto a concave slide and 0.9% glycerol (g9012, Sigma Aldrich) with 0.1% N-propylgallate (MKCC 1933, Sigma Aldrich) in PBS and was used as anti-fade, followed by sealing with a cover glass.

For retinal cryosections, the posterior segments were embedded in increasing levels of sucrose from 5% to 30% in PBS. Samples were then mounted in O.C.T. Compound (Tissue-Tek^®^ O.C.T. Compound, #4583) and then frozen with dry ice and kept at −80°C for at least 24 h. Then, 10–20 μm cryosections were obtained at −22°C (Leica, CM 1950, Switzerland).

Next, slides were washed with PBS and 0.5% PBS-TWEEN. After 1 h of incubation in blocker solution, slides were incubated overnight at 4°C with the primary antibodies (see Table 3, Supplementary Material), except for the ChAT staining, which required a long incubation time of three nights. Next, slides were washed with PBS and incubated for 1 h at room temperature with secondary antibodies (see Table 4, Supplementary Material). Slides were washed with PBS and then stained with Hoechst 1:1,000. Finally, anti-fade was used, followed by sealing with a cover glass. The ready slides were shielded from light and maintained at 4°C. Images were obtained using Leica confocal Live and Stellaris confocal microscopes and were used to count the cell number in the ONL, at the time points of interest.

### *In-vivo* characterization

#### OCT imaging

GCaMP6f^+\−^RCS^-\-^ rats were anesthetized with i.m 1 mg/kg ketamine (Ketamidor, Richter Pharma) and 0.5 mg/kg xylazine (Sedaxylan, Eurovet Animal Health) after which the pupils were dilated. Optical coherence tomography images (OCT, Spectralis, Heidelberg) were acquired and served to measure the thickness of the various retinal layers (ONL, ONL + OPL + debris, INL, and whole retina), as a part of a long-term follow-up (4, 8, and 12 weeks). In addition, confocal scanning laser ophthalmoscopy (cSLO) fluorescence imaging was performed to visualize the fluorescence induced by GCaMP6f gene expression in the RGCs.

#### *In-vivo* ERG recording

Focal electroretinogram (fERG) recordings were performed using the Micron IV (Phoenix Research Laboratories, Inc.) equipped with a focal ERG stimuli module; the electrophysiological response was recorded using the AlphaSNR recording system (Alpha Omega Ltd., Israel), similar to our previous publications (Arens-Arad et al., 2020; Arens-Arad et al., 2021). Animals were anesthetized and the pupils were dilated with Mydramide (0.5% Tropicamide, Fisher Pharmaceuticals, Ltd.) and Efrin-10 (10% Phenylephrine HCL, Fisher Pharmaceuticals Ltd.) drops. For ERG recordings, the cornea was brought into contact with the Micron IV imaging lens, which served as the recording electrode; a reference electrode was placed on the animals’ nose, and a ground electrode was placed in the animals’ tail. Stimuli (2 mm in diameter) were projected with intensities ranging from 0.00978 to 640 cds/m^2^ with a pulse duration of 2 msec at 2 Hz. The acquired ERG signals were recorded for over 60 s and averaged and analyzed offline using a custom-written MATLAB program (The Mathworks, Waltham, MA, United States).

### *Ex-vivo* RGC calcium imaging recordings

To demonstrate the applicability of the novel animal model in the broader context of vision restoration and specifically in studying electrical stimulation of the retina, we focused on determining the thresholds for subretinal electrical stimulation in isolated retinas at various stages of degeneration (4, 8, and 12 weeks). These thresholds were then compared with those from control retinas, which were isolated from Long Evans GCaMP6f animals (LETg (Thy1-GcaMP6f)7). In addition, we studied the spatial spread of a single electrode activation at various stimulation currents.

#### Retinal tissue preparation

The animals were euthanized using CO_2_ inhalation, after which the eyes were enucleated and hemisected in Ringer’s solution (110 mM NaCl, 22 mM NaHCO_3_, 2.5 mM KCl, 1.6 mM MgCl, 1 mM CaCl_2_, NaH_2_PO_4_, and 10 mM Glucose) and then continuously oxygenated with 95% O_2_ and 5% CO_2_. The vitreous humor and lens were carefully removed from the opened eyecup using precision forceps. The retina was then hemisected and gently separated from the retinal pigment epithelium. The isolated rat retina was then mounted, with the photoreceptor side down, on a Multi-Electrode Array (MEA- 60MEA200/30iR-Ti-pr-T, Multi-Channel System Germany) for electrical stimulation via a current injection system (MEA 2001, Multi-Channel System Germany). Cathodic pulses ranging from 0.1 to 1 ms and 10–120 µA were applied at a frequency of 0.2 Hz, and the induced RGCs responses were analyzed.

#### Calcium imaging

The electrically induced RGCs responses were visualized using an upright microscope (Slicescope 6,000, Scientifica, Uckfield, United Kingdom) equipped with a CCD camera (EXI-Blue QIMAGING) and a filter set (EX 488 nm/EM 525 nm) to allow for fluorescent image acquisition at 10 frames per second. The activation threshold was assessed from the change in the fluorescence signal from the baseline using a custom-written MATLAB code, which was previously described by our group (Schick et al., 2020; Shpun et al., 2023). Briefly, as a first step, the change in the fluorescence frame was calculated, pixel by pixel, by subtracting each acquired camera frame from the average baseline frame (calculated as the mean stimulus-free frames). Next, the change in percentage for each frame was calculated by dividing the subtraction product by the average baseline frame. To evaluate the change in the cell of interest, the fluorescence change in the relevant pixels was averaged. The average fluorescence change was then corrected for photobleaching by fitting the data to a two-time constant exponent and subtracting the obtained fit from the data.

To evaluate the spatial spread of the electrically induced activation, z-score maps (a standard deviation normalization of the fluorescence change in each pixel) were constructed, and the diameter of the activated area was estimated by fitting a 2D Gaussian to pixels with a z-score higher than 2. This area was then fitted to a Gaussian distribution, and the average FWHM served as a measure of the spread radius.

## Results

### Establishing the model

**FIGURE 1:**
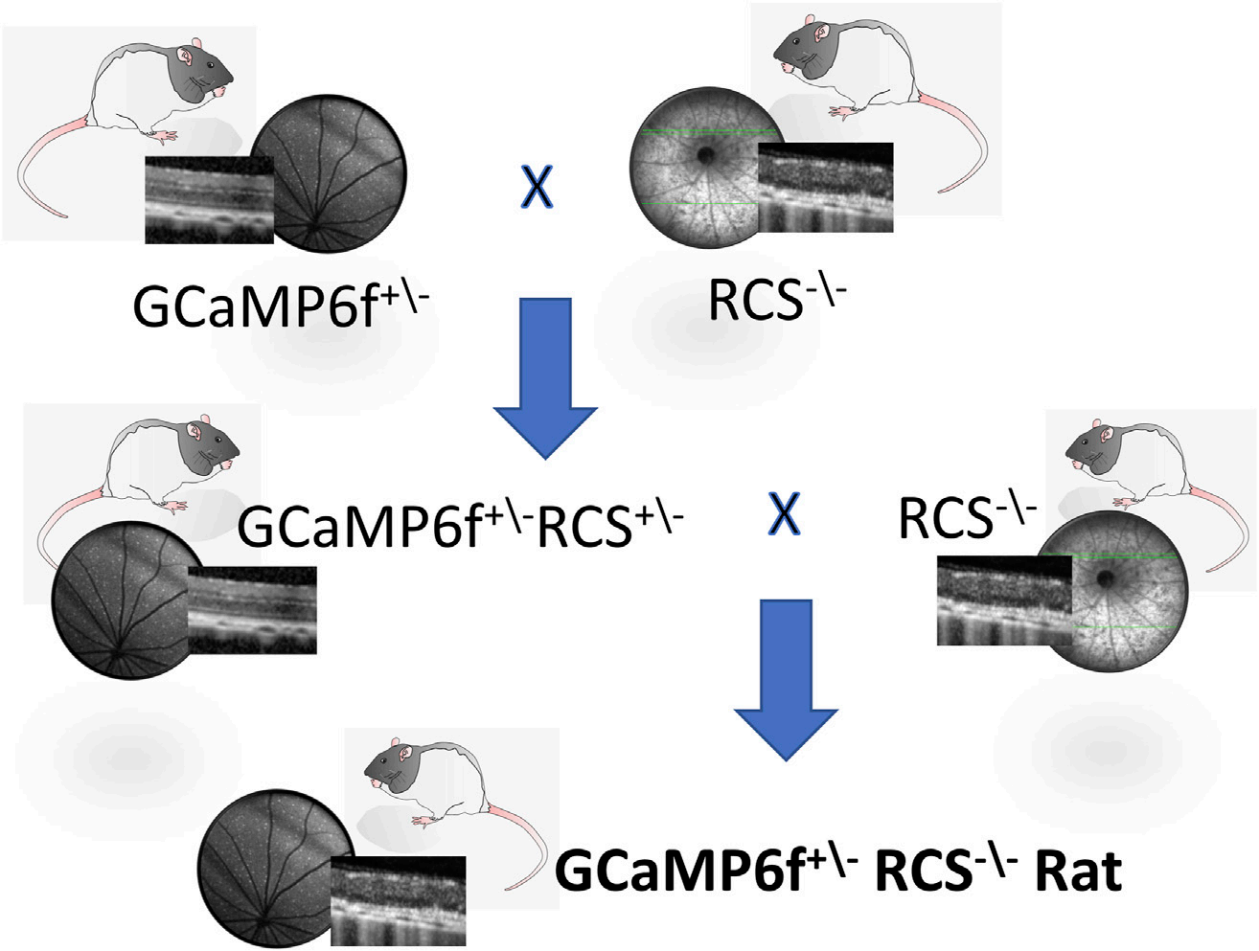
The GCaMP6f^+\-^ RCS^-\-^ rat breeding scheme. The GCaMP6f^+\−^RCS^-\-^ Rat, an RCS with GCaMP6f positive retinal ganglion cells, is obtained through the cross-breeding of the RCS^−\-^ (MERTK^−/−^) rat with a Long Evans (LE) hemizogtyc GCaMP6f^+\-^. This is a two-generation breeding process, first yielding a GCaMP6f^+\−^RCS^+\-^, with the RCS mutation on a single allele. Breeding the first generation with an RCS^−\-^ yields the desired breed (25%).

As described in the Methods section, the GCaMP6f-RCS rat model was obtained following a two-generation crossbreeding (Figure 1). This was then validated through gel electrophoresis where the expected bands for the genes of interest were observed (Supplementary Figure S1S, Supplementary Material) (750bp double alleles for the RCS mutation, 609bp for the WT allele in heterozygotic RCS rats and 248bp for the GCaMP6f). Further histological, anatomical, and functional evaluations of the retina were performed as detailed next.

### GCaMP6f is robustly expressed in retinal ganglion cells

**FIGURE 2:**
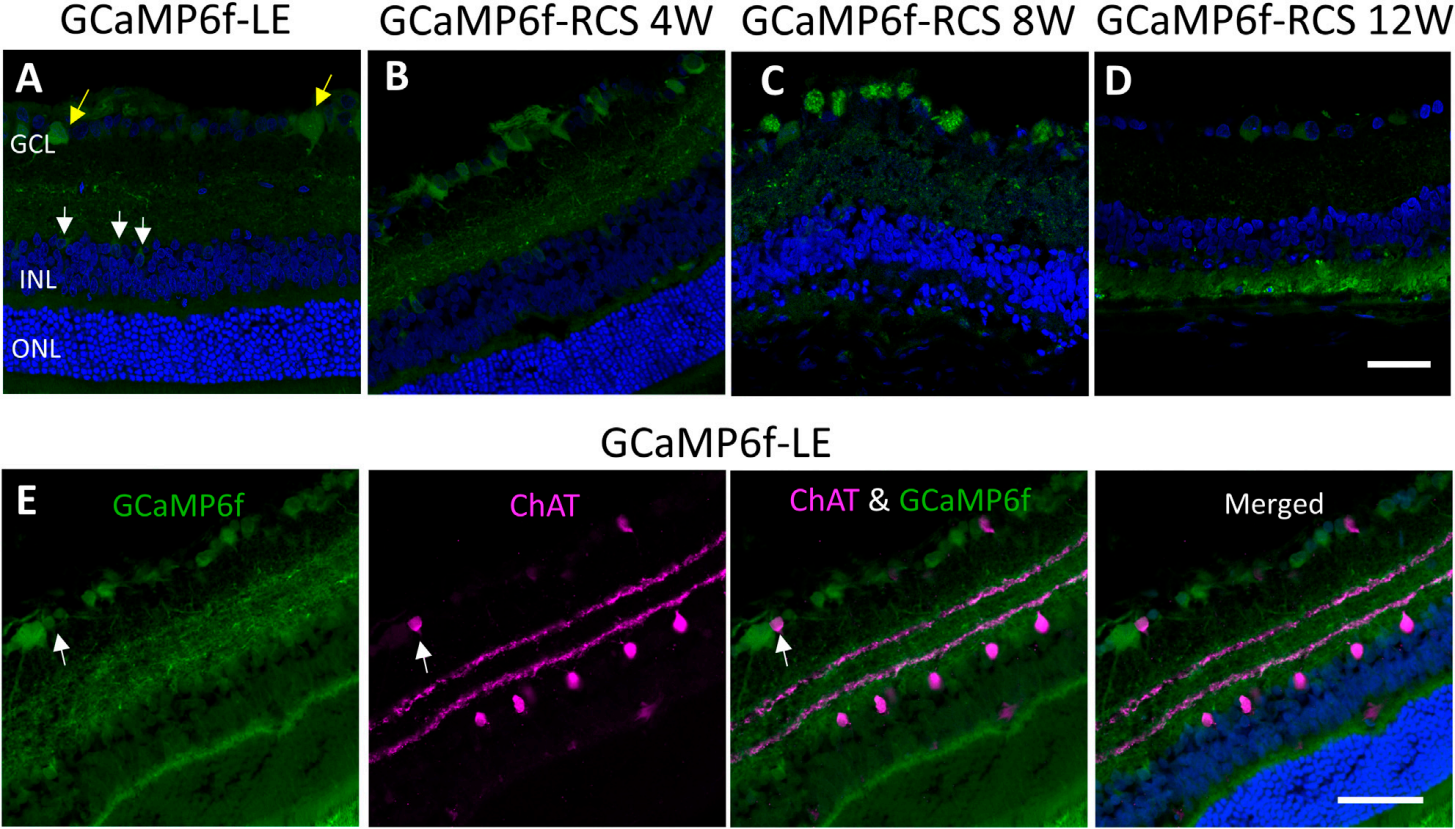
Studying GCaMP6f expression in different retinal layers. **(A)** Retinal section of a GCaMP6f-LE retina with nuclei staining (Hoechst) highlighting the expression of GCaMP6f in the RGC layers (yellow arrows) and a few positive cells in the INL (white arrows). **(B–D)** The staining in GCaMP6f-RCS retinas throughout the degeneration process demonstrates the preservation of the GCaMP6f expression pattern. **(E)** Staining of GCaMP6f-LE retina with the cholinergic amacrine marker (ChAT) showing no co-localization with GCaMP6f, neither in the INL nor the RGC layer. Scale bar - 50 µm.

We thoroughly investigated the expression of GCaMP6f in the different cell types and the retinal layers of both GCaMP6f-LE and the obtained (GCaMP6f-RCS) rats. To this end, histological retinal sections were prepared from eyes enucleated from GCaMP6f-LE and GCaMP6f-RCS rats at the different time points of interest (4, 8, and 12 weeks) and imaged using confocal microscopy (see the Methods). Imaging revealed that GCaMP6f expression was almost exclusively localized to the RGCs layer in both the LE and the obtained RCS breeds (Figures 2A–D, yellow arrows). In the RGCs layer, 11.5% of GCaMP6f positive cells co-expressed ChAT (Figure 2E, white arrows), suggesting that they are displaced amacrine cells. These cells were morphologically smaller, exhibiting a weak fluorescence signal (similar to observations made by Raymond et al. (2008)).

Only a small percentage (3.18%) of INL cells express GCaMP6f (white arrow). These INL cells did not co-express ChAT (Figure 2E), Recoverin, or PKC-alpha (Supplementary Figure S2S), suggesting that they are neither cholinergic Amacrine, cone bipolar, nor rod bipolar cells, respectively.

**FIGURE 3:**
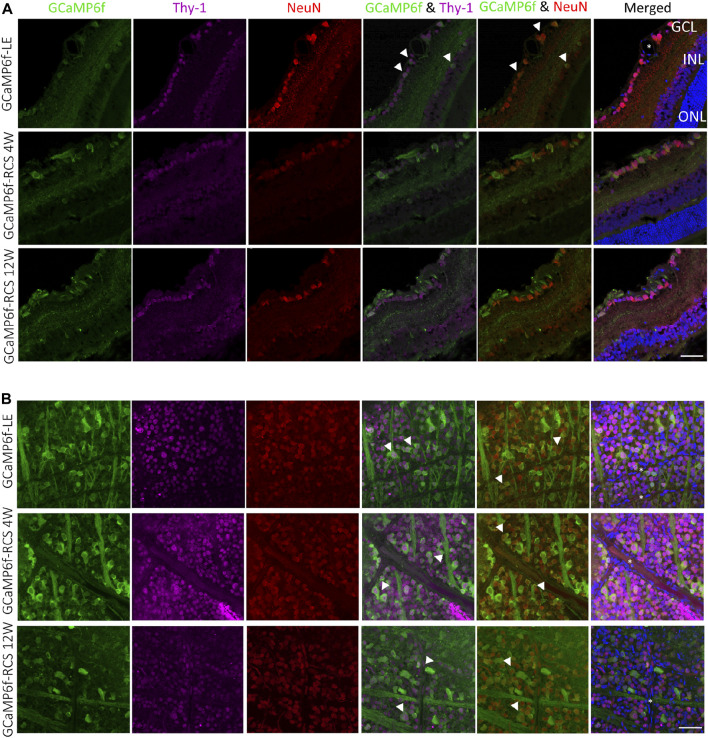
Histological investigation of neuronal and RGC markers’ co-localization with GCaMP6f. **(A)** Sections of GCaMP6f-LE and GCaMP6f-RCS positive retinas stained for both Thy-1 (the promotor under which the GCaMP6f is expressed) and NeuN (the neuronal nuclei antibody), a neuronal marker for the investigated age groups. **(B)** Wholemount GCaMP6f-LE and GCaMP6f-RCS positive retinas stained for both Thy-1 and NeuN. These images were used to identify the GCaMP6f positive cells and to quantify the co-localization (white arrowheads) of GCaMP6f with the various retinal cell types. *Blood vessels. Scale bar - 50 µm.

To further study the identity of GCaMP6f expressing cells, retinal sections (Figure 3A) and whole mount retinas (Figure 3B) were stained for Thy-1 (the promotor under which the gene is expressed and a putative marker for RGCs and amacrine cells) and for NeuN (neuronal nuclei antibody, a neuronal marker known to be expressed in RGCs (Wolf et al., 1996; Wang et al., 2000)). Retinal sections show that most cells co-expressed GCaMP6f, Thy-1, and NeuN (white arrowheads). Using the wholemount samples, we found that in the RGCs layer of GcaMP6f-LE, 90.9% and 87% of the GcaMP6f expressing cells co-expressed Thy-1 and NeuN, respectively; these results suggest that the GcaMP6f cells are almost all RGCs. Interestingly, 76.9% of the cells expressing Thy-1 and 95.7% of the cells expressing NeuN co-expressed GCaMP6f, suggesting a high penetrance of the selected Thy-1 promoter. Overall, 67% of the cells in the RGCs layer expressed GCaMP6f, in agreement with (Raymond et al., 2008). It should be mentioned that GCaMP6f is not a stable fluorescence marker, but rather a calcium indicator, thus not all GCaMP6f RGCs were counted. We therefore further stained retinal sections with anti-GFP antibody, and indeed found that about 22% of RGC-GFP positive cells were not exhibiting GCaMP6f. (Supplementary Figure S3).

These values were relatively preserved in the GCaMP6f-RCS rat; at the age of 12w, 100% of the cells expressing Thy-1 and 91.3% of the cells expressing NeuN co-expressed GCaMP6f; all cells that expressed GCaMP6f in the RGCs layer also co-expressed either NeuN or Thy-1.

### GCaMP6f-RCS exhibited a gradual retinal degeneration

**FIGURE 4:**
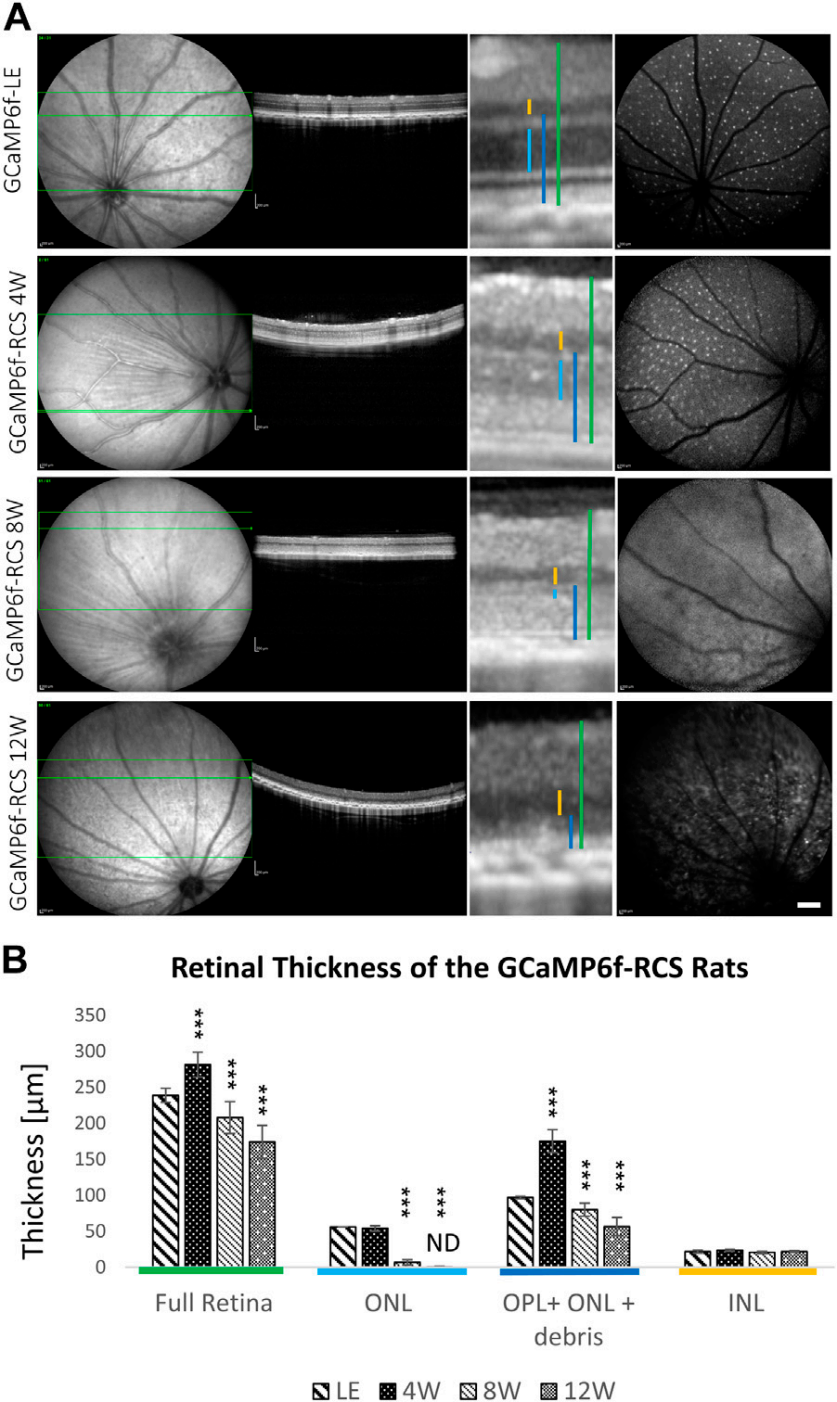
Progressive loss of outer retinal layers in the GCaMP6f-RCS rat. **(A)** Representative cSLO and OCT images of a control GCaMP6f-LE animal and GCaMP6f-RCS rats at the different age groups. **(B)** Quantification of different retinal layers’ thickness throughout the degeneration process of GCaMP6f^−^ RCS compared to the GCaMP6f-LE animal. Imaging revealed a gradual thinning of the outer retinal layers, in line with the histological studies. ONL, outer nuclear layer; OPL, outer plexiform layer; INL, inner nuclear layer. Scale bar - 1 mm.

The progression of retinal degeneration was verified and quantified using two different methods. The first relied on quantifying the thickness of the various retinal layers in the acquired OCT images (Figures 4A, B). A progressive decrease in the ONL thickness was observed (p for trend <0.0001) throughout the degeneration process in the GCaMP6f-RCS rat ONL, with no ONL detected in OCT images by 12w of age. A similar decrease in full retinal thickness and OPL + ONL + debris was found throughout the degeneration process. In contrast, the INL thickness was preserved (*p* = 0.18). It should be noted that the total retinal thickness for the 4 W GCaMP6f-RCS group was larger than that of the wild-type rats, probably because of the debris layer (Figure 4B). Interestingly, cSLO imaging showed a sparse appearance of the RGCs in the WT and RCS at 4w, whereas in the older RCS rats the RGCs fluorescence was obscured by the autofluorescence of the degenerated retina.

**FIGURE 5:**
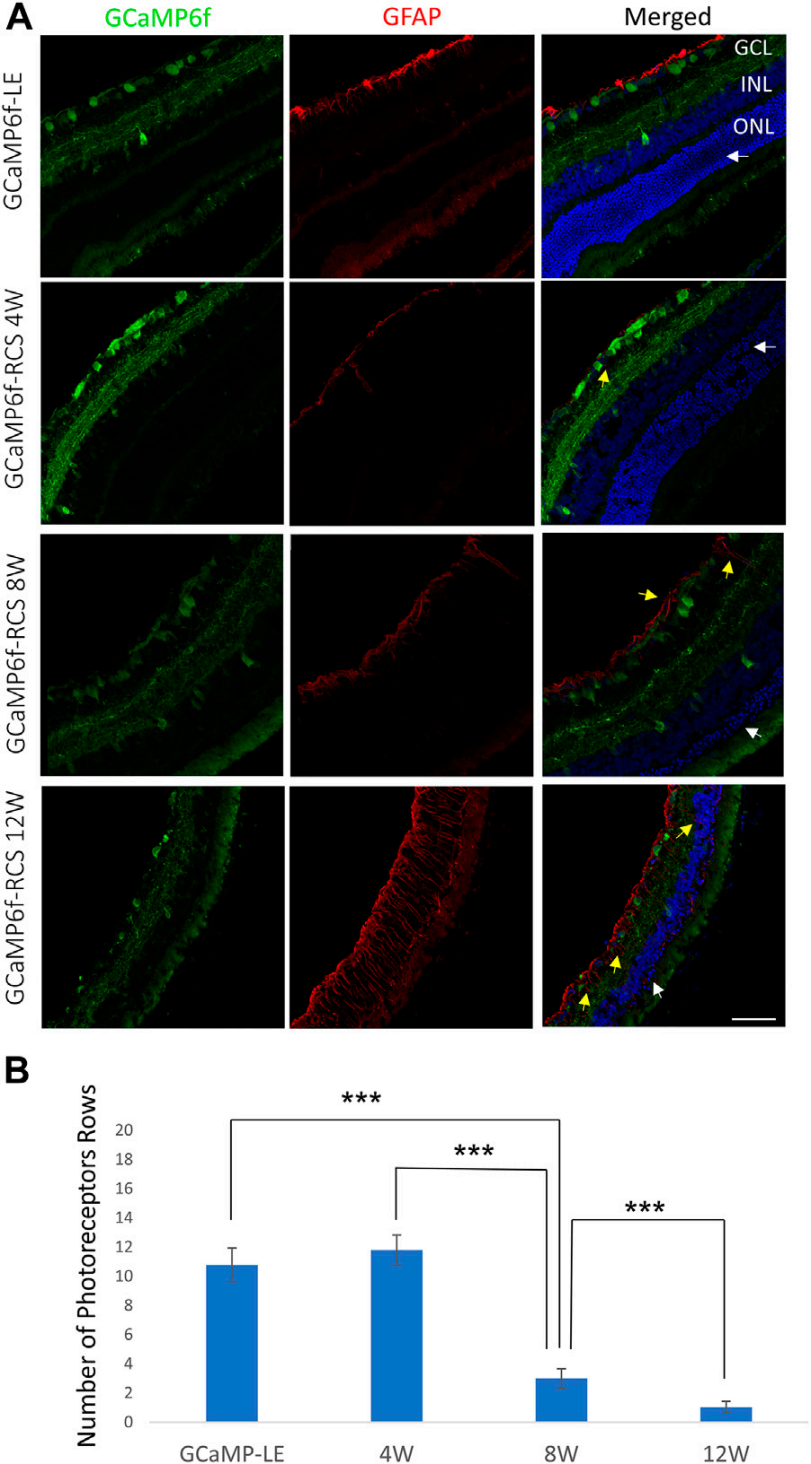
Progressive loss of photoreceptors in the GCaMP6f-RCS rat. **(A)** Retinal histological sections of the GCaMP6f-LE and GCaMP6f-RCS rats throughout the degeneration process were stained for Hoechst and fibrillary acidic protein (GFAP). The sections show a significant loss of photoreceptors in the ONL throughout the degeneration process. In addition, an increase in the activated glial marker (GFAP) was observed. **(B)** The number of photoreceptor rows quantified at the various investigated time points compared to control GCaMP6f-LE animals revealed a significant decline in the number of photoreceptor rows throughout the degeneration process. ***t-test *p* < 0.001. Scale bar - 50 µm.

In agreement with the OCT imaging, histological sections from 4, 8, and 12-week-old GCaMP6f^+\−^RCS^-\-^ rats showed a progressive decrease in the thickness of the retinae throughout the degeneration process (Figure 5A). Quantification of the number of photoreceptor nuclei in the ONL (white arrow) showed a significant decrease throughout the degeneration process (10.76 ± 1.16, 11.79 ± 1.03, 3.00 ± 0.67, and 1.03 ± 0.39 for WT, 4w RCS, 8w RCS (*p* < 0.005), and 12w RCS (*p* < 0.005), respectively (Figure 4B). In addition, increase in the GFAP staining levels (yellow arrow) were observed throughout animal aging, suggesting an active degeneration and remodeling, as previously reported (Zhao et al., 2010) (Figure 5A).

### GCaMP6f-RCS rats exhibited a gradual loss of visual function

**FIGURE 6:**
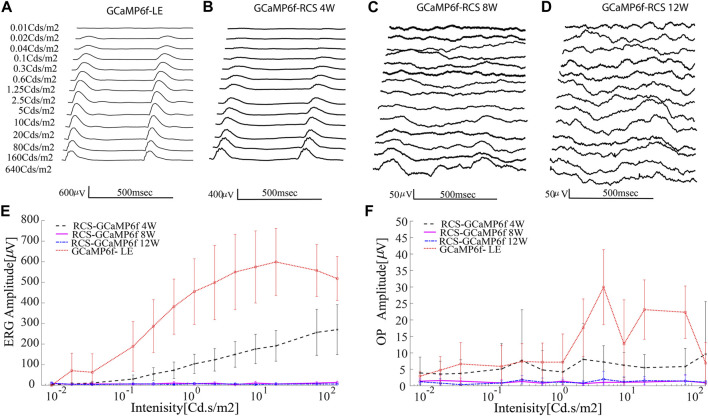
Progressive loss of retinal function in the GCaMP-RCS. **(A–D)** Representative average ERG signals for increasing luminance for the various animal groups. **(E)** The average ERG amplitude (defined as the difference between the maximum and the minimum of the average signal) as a function of intensity revealed a diminished ERG signal throughout retinal degeneration. No ERG signal was recorded at the age of 12w. **(F)** The average OP amplitude (defined as the difference between the maximum and the minimum of the average OP signal) as a function of intensity reveals the diminished ERG signal throughout degeneration.

As part of characterizing the retinal degeneration of GCaMP6f-RCS rats, we investigated the ERG amplitude at different age groups (n-8, n = 8, n = 6 for 4W, 8W and 12W respectively) and compared it to that of a control group of GCaMP6f-LE animals (n = 5). Figure 6 illustrates representative acquired ERG signals (Figures 6A–D) and an average ERG amplitude-intensity curve (Figure 6E) obtained for each animal group. The results show a significant decrease in ERG amplitude with degeneration (p for trend *p* < 0.001), whereby by 12 weeks, no signal can be observed even for the highest investigated stimulus intensity. These results are in agreement with previous reports (Cuenca et al., 2005; Adachi et al., 2016) and with the histologically observed extensive PR degeneration (Figure 5). An additional component of interest in the ERG signal is the Oscillatory potentials (OPs), which can be isolated by applying a bandpass filter in the range of 80–160 Hz (representative OPs for 80Cds/cm^2^ are presented in Supplementary Figure S4). Figure 6F presents the peak of these oscillatory potentials as a function of intensity for the animal groups, revealing a trend similar to that of the ERG amplitude, in which this component decreases with the maturation of the dystrophic animal, in agreement with previous reports (Cuenca et al., 2005).

### Optical recording of ganglion cell responses to electrical stimulation of GCaMP6f-RCS retina

**FIGURE 7:**
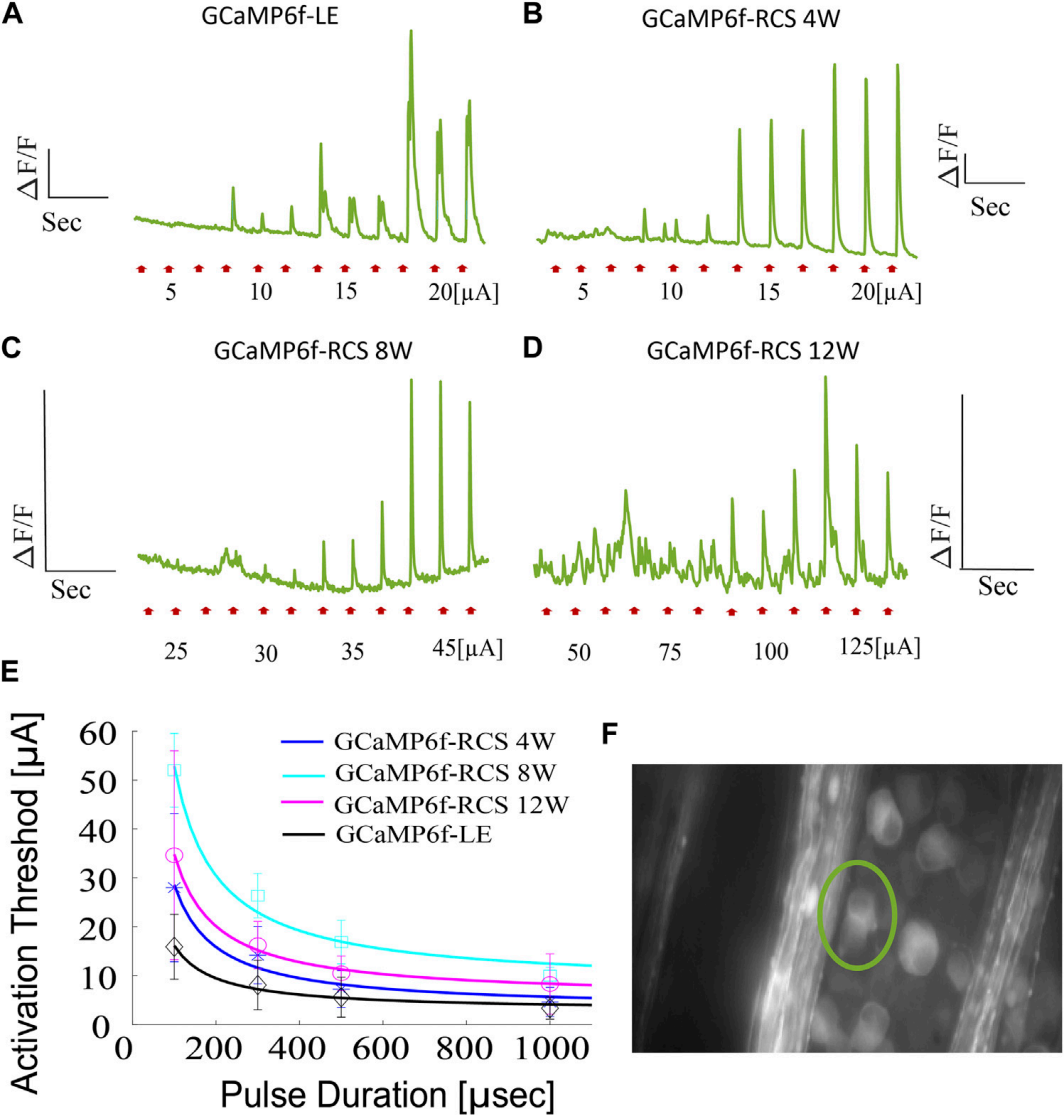
*Ex-vivo* subretinal stimulation of GCaMP6f^+\-^ RCS^-\-^ rat retina. **(A–D)** Representative average fluorescence signal changes induced by a 100µsec pulse with increasing current amplitude utilized in investigating the activation in GCaMP-LE, 4W, 8W, and 12W GCaMP- RCS rats, revealing activation thresholds of 10, 15, 35, and 100 µA, respectively. **(E)** The average activation threshold as a function of pulse duration (strength-duration curve) for the various investigated groups (the solid line is the Lapique fit), highlighting the expected increase in activation threshold throughout the degeneration process. **(F)** A representative FOV, the area for which the average fluorescence change was calculated, is demarcated.

As an example of utilizing the novel developed breed for optical recording of retinal function, we investigated the electrical stimulation of isolated retina using a commercially available MEA (see the Methods). The isolated retina was stimulated at various pulse durations with increasing current amplitude while recording the RGCs calcium signals (presented as a change of fluorescence signal from baseline, dF/F). The stimulation threshold was then defined as the current amplitude that induced a robust (>2 STDEV) change in the fluorescence signal from the baseline Figures 7F, is a representative fluorescence image with the pixels of interest marked. Figures 7A–D depicts representative RGCs calcium signals induced by 100µsec current pulses for the different investigated age groups showing increasing calcium responses with increasing stimulation current amplitude. Stimulation thresholds for 100µs pulses were found to be 15.9 ± 6.64 (*n* = 11 cells), 25.625 ± 13.2 (*n* = 10), 52 ± 7.52 (*n* = 11), and 34.61 ± 21.35 µA (*n* = 13) for LE, RCS 4W, 8W, and 12w, respectively (*p* = 0.038 for LE vs. RCS-8W, ANOVA1 for age: *p* < 0.0001, p for trend: <0.001).

Activation thresholds were further measured at various pulse durations to create a strength-duration curve, which further demonstrated the increase (*p* < 0.0001) in the current threshold throughout the retinal degeneration process (Figure 7E).

**FIGURE 8:**
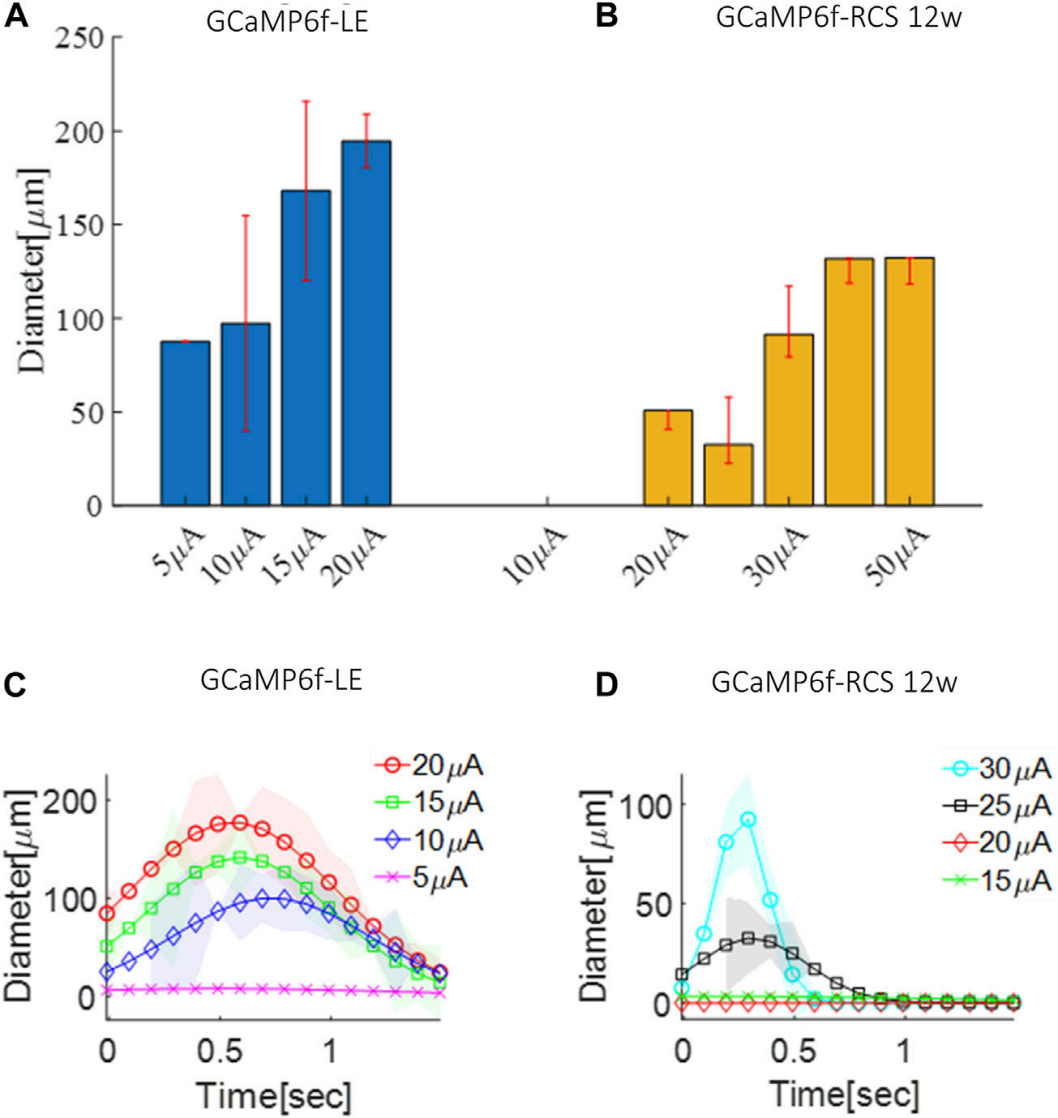
Spatial spread of the activation area in GCaMP6f^+\-^ RCS^-\-^ rat retina. **(A)** The average diameter of the activation area as a function of the current amplitude for a GCaMP-LE rat. **(B)** The average diameter of the activation area as a function of the current amplitude for a 12 W GCaMP-RCS rat. **(C)** Time dynamics of the activation area diameter for an increasing current in a GCaMP-LE rat. **(D)** Time dynamics of the activation area diameter for an increasing current in 12 W GCaMP-RCS rats.

Next, we demonstrated the applicability of this animal model for evaluating the retinal activation area diameter induced by subretinal stimulation, through a 30 µm electrode, for a 100 µsec pulse with various current amplitudes using the data acquired as part of the threshold investigations described above. To this end, we constructed the z-score maps of the acquired calcium imaging and estimated the spread (see the Methods). Estimation of the activation area diameter for a 100µsec pulse at the threshold current revealed a diameter of 105µm and 60 µm for the GCaMP6f-RCS and 12 W GCaMP6f-RCS rats (*p* = 0.0082), respectively (Figures 8A, B), comparable to (Tong et al., 2020). Increasing the stimulation currents resulted in a significant increase in the spatial spread of the activated area for both breeds (Figures 8A, B). Interestingly, fitting the activation area diameter for the threshold current to a time Gaussian revealed that the activation dynamics were faster in the GCaMP6f-RCS rats (0.59 ± 0.76 s) compared with the GCaMP6f-RCS rats (0.12 ± 0.11), *p* = 0.036 (Figures 8C, D).

## Discussion

Research into vision restoration for retinal degenerative diseases heavily relies on the ability to investigate the responses of retinal ganglion cells (RGCs) induced by prosthetic activation, optogenetics, or cell replacement therapy. We introduced here a novel animal model of the well-known retinal degenerated RCS rat that further expresses the calcium indicator GCaMP6f in the RGCs, thus enabling the *ex-vivo* and *in-vivo* optical investigation of RGCs following prosthetically induced activation. Although optical monitoring of RGCs activity has been previously reported (both *ex vivo* (Weitz et al., 2013) and *in vivo* (Li et al., 2022)), it was obtained mainly through the viral transfection of the GECI in the RGCs, thus limiting its applicability; therefore, it is not widely used in RD rodent models. The rat has long been the animal of choice for investigating vision restoration strategies: its eye dimensions enable implanting various retinal prostheses (Mathieson et al., 2012; Chang et al., 2019; Arens-Arad et al., 2020; Shpun et al., 2023) and investigating various electrical stimulation strategies. Optimization of the electrical stimulation parameters and the configuration was also extensively studied in isolated rat retinal preparations (Sekirnjak et al., 2006; Boinagrov et al., 2014). The utilization of the rat retina as a model has been further driven by the abundant available data on its characteristic responses to natural visual stimulation in both normal and degenerated retina (Wong et al., 2012; Yu et al., 2017).

The successful preservation of the RD features of the RCS rat and the expression of GCaMP6f in the RGCs were extensively validated in the newly developed model. A detailed verification of the identity of the cells exhibiting GCaMP6f fluorescence was obtained through histological investigations, similarly to (Raymond et al., 2008). Immunohistochemistry studies confirmed that about 90 percent of GCaMP6f-expressing cells are RGCs; other cells might be displaced amacrine cells, which can be inferred by the ChAT staining; these findings are in agreement with (Raymond et al., 2008).

The retinal degeneration process in the new breed resembled that of the RCS rat (Ryals et al., 2017b), as evident from the gradual thinning of the ONL and the accumulation of subretinal debris, observable in OCT imaging and histology; the thickness of the other retinal layers was preserved (Ryals et al., 2017b). The RCS retinal degeneration features were further confirmed through functional evaluation of ERG signals, showing the complete cessation of ERG signals by 12 weeks, in agreement with previous reports (Cuenca et al., 2005).

Next, we demonstrated the applicability of this novel animal model as a tool in the field of retinal prostheses by investigating subretinal electrical activation thresholds *ex vivo*. The electrical activation thresholds, measured by the optical methods, were similar to previous reports (Jensen, 2012; Boinagrov et al., 2014). More importantly, we found a gradual increase in the activation threshold throughout the retinal degeneration process, in line with previous reports (Ren et al., 2018; Tong et al., 2020). The use of this animal model enables an easy and robust characterization of the current threshold and the strength-duration curve in any electrode geometries [e.g., (Bhuckory et al., 2023; Shpun et al., 2023)], or current injection protocols.

Investigating the extent of the spatial activation spread is another crucial factor in determining the expected obtained resolution of a specific set of electrode geometries (Butterwick et al., 2009; Flores et al., 2019) or a stimulation paradigm (e.g., current steering (Dumm et al., 2014; Khalili Moghaddam et al., 2014; Wang et al., 2022)). We demonstrated that our novel model can readily contribute to these efforts by investigating the diameter of the activated area following electrical stimulation. We also observed that with increasing stimulation current, the spatial extent of stimulation significantly increased, similar to (Tong et al., 2020). Interestingly, we found that the spatial extent of activation in degenerated rats was smaller compared with WT.

Notwithstanding its many merits, such as its high throughput, the feasibility of monitoring the activity at single cell resolution, the simplicity of simultaneous subretinal stimulation and optical recording of the induced response, and the possibility to perform *in-vivo* studies (Li et al., 2022) the use of this retinal model is not without its limitations. The first is that a minority of the putative RGCs calcium signals may have originated from amacrine cells, which can be inferred from the GCaMP6f expression in some ChAT-positive amacrine cells. More importantly, using calcium dynamics to infer neural activity at the single action potential resolution necessitates optimal imaging conditions (even using the two-photon imaging technique), thus limiting the field of view (Wei et al., 2020; Huang et al., 2021).

To conclude, the animal model presented here can serve as a vital tool for studying the activation of the retina by electrical or other stimulation methods. This provides an opportunity to explore and enhance our understanding of retinal responses, potentially leading to significant advancements in prosthetic interventions for visual impairments.

## Data Availability

The raw data supporting the conclusion of this article will be made available by the authors, without undue reservation.
