# Supplementary material for: A novel GCaMP6f-RCS rat model for studying electrical stimulation in the degenerated retina
Source: Front Cell Dev Biol. 2024 Apr 22;12:1386141. doi: 10.3389/fcell.2024.1386141 (PMC11070775; doi:10.3389/fcell.2024.1386141)
Supplement: Supplementary file 4 [file DataSheet1.docx]

Supplementary Material

**Novel GCaMP6f-RCS Rat model for studying electrical stimulation in the degenerated retina**

Tamar Azrad Leibovitch^1,2†^, Nairouz Farah^1,2†^, Amos Markus^1,2^, Yossi Mandel*^1,2^

1. Bar Ilan Institute for Nanotechnology & Advanced Materials (BINA), Bar Ilan University, 5290002, Ramat Gan, Israel.

2. Faculty of Life Sciences, School of Optometry and Vision Science, Bar Ilan University, 5290002, Ramat Gan, Israel.

*** Correspondence:**Corresponding Author
[yossi.mandel@biu.ac.il](mailto:yossi.mandel@biu.ac.il)

† These authors contributed equally

**Primers and genotyping**

**Table 1** **– Primers used in the PCR genotyping.**

Genotyping for the validation of the animal genome across various generations of the breeding process was achieved using the primers detailed in Table 1.

|  | Primers |
| --- | --- |
| (1) Primer 3625 (RCS) | CACTCTCTGGTAGCCATTG |
| (2) Primer 3741 (RCS) | TGGGACTAGCCTCAGTTCAC |
| (A) 3741 related (RCS) | ATGAACGAGGCAGGAAATGT |
| GCaMP F (GCaMP6f) | CATCAGTGCAGCAGAGCTTC |
| WPRE R (GCaMP6f) | CAGCGTATCCACATAGCGTA |

**Table 2 – PCR protocol optimization**

PCR was optimized for the primers at hand as described in Table 2.

| GCaMP6f | RCS |
| --- | --- |
| 95^o^C 3 minutes | 94^o^C 3 min |
| 94^o^C 30 seconds | 94^o^C 30 seconds |
| 62^o^C 30 seconds | 61^o^C 30 seconds |
| 72^o^C 30 seconds - Repeat steps 2-4 34 times for a total of 35 cycles | 72^o^C 3 min - Repeat steps 2-4 29 times for a total of 30 cycles |
| 72^o^C 10 minutes | 72^o^C 4 minutes |
| 4^o^C hold | 4^o^C hold |

**Histological Investigations**

**Table 3** **- Retinal cells primary antibodies.** The various retinal cell types were identified by staining either whole mount retinae or cryosections for the following primary antibody concentrations.

|  | Host | Concentration | Cat# |
| --- | --- | --- | --- |
| GFAP | Mouse | 1:400 | 73-240, NeuroMab |
| PKCa | Rabbit | 1:250 | P4334, Merck |
| Recoverin | Rabbit | 1:200 | AB5585, Merck |
| Thy-1 | Rabbit | 1:200 | BS-0778R, Boiss |
| NeuN | Mouse | 1:200 | MAB377, Millipore |
| ChAT | Goat | 1:200 | AB144P, Sigma Aldrich |
| anti -GFP | Rabbit | 1:200 | SC-8334 Santa Cruz |

**Table 4** **- Secondary antibodies for Immunofluorescent staining.** The following secondary antibodies were used.

|  | Contrarotation |  |
| --- | --- | --- |
| Donkey anti Rabbit 647 | 1:200 | AB-ab150075, Abcam |
| Donkey anti Rabbit 594 | 1:200 | AB_162543, Invitrogen |
| Donkey anti Mouse 647 | 1:200 | AB_2340854, Abcam |
| Donkey anti Goat 647 | 1:200 | Ab150131, Abcam |
| Donkey anti Rabbit 594 | 1:200 | 711-585-152 Jackson |

**Electrophoresis genotyping**

Gel electrophoresis was performed to validate the genotype of the newly obtained breed. The expected bands for the genes of interest were observed.


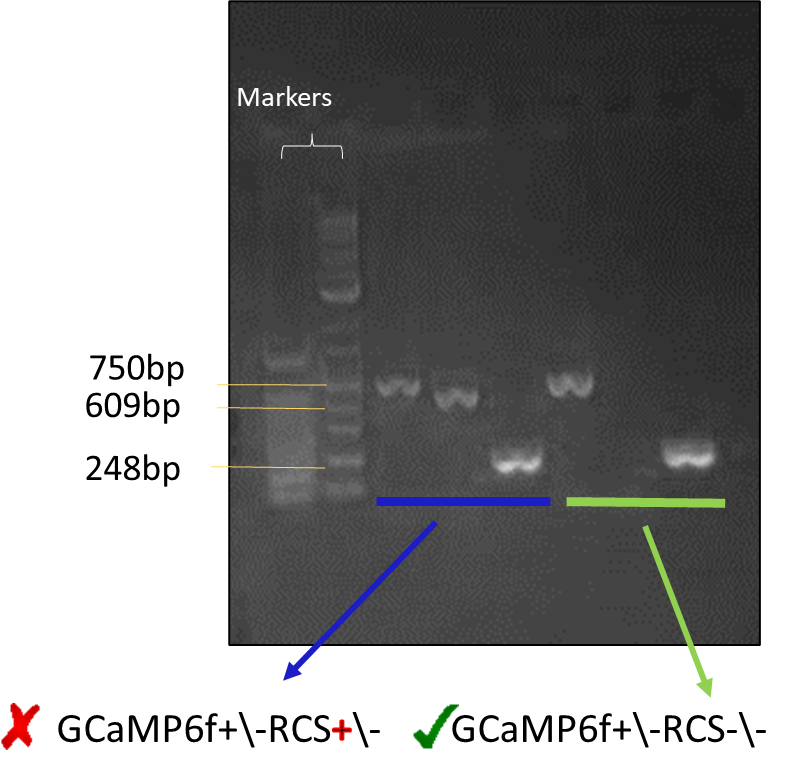


**Figure1 Supp.** **The novel breed Genotyping**. Electrophoresis to identify the genotypes of the animal. In blue- The GCaMP6f^+\-^RCS^+\-^ Rat. In green- GCaMP6f^+\-^RCS^-\-^ Rat. 750bp are double alleles for the RCS mutation, 609bp for WT allele in heterozygotic RCS rats and 248bp for the GCaMP6f.

**GCaMP6f expression is mainly localized to the RGC layer.**

To study the localization of the GCaMP6f to the RGC layer, we stained retinal sections (as described in the methods) with the rod bipolar cells marker- PKCa and the cone bipolar marker Recoverin, to rule out GCaMP expression in the bipolar cells situated in the INL. Our results reveal (Supp. Fig2) that the GCaMP6f expression is localized to the RGC layer with very few cells positive cells in the INL (3.29± 1.86%).

GCaMP6f-LE


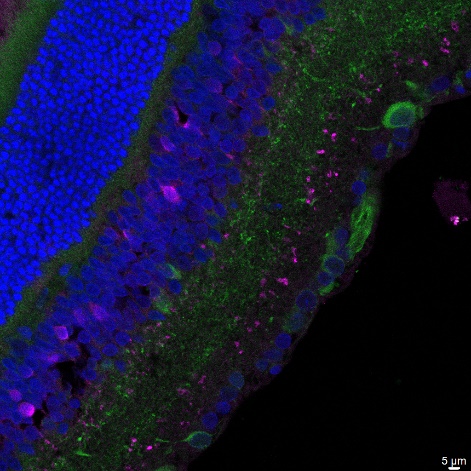

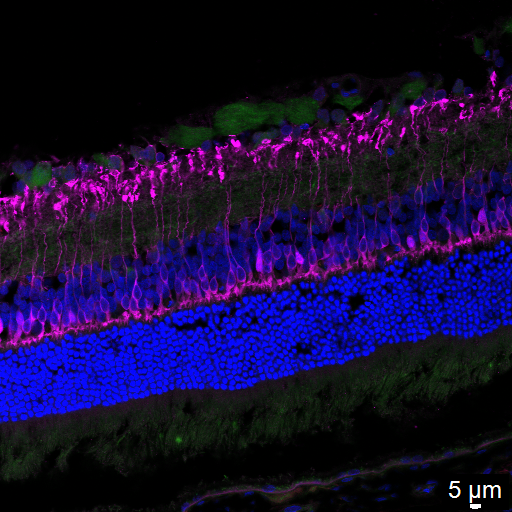


PKCα

Recoverin

**Figure 2 Supp. Cell type characterization of the GCaMP6f-LE**. Staining for Recoverin (a cone bipolar marker) and PKCα (rod bipolar cells marker) highlighting the lack of overlapping with GCaMP6f positive cells. Scale bar 25µm.

**Anti-GFP staining for the validation of GCaMP6f positive RGCs counting.**

We stained retinal sections with anti-GFP antibody (as described in the methods), and observed that only about 22% of RGC-GFP positive cells did not exhibit GCaMP6f fluorescence. (Supp Fig. 3).


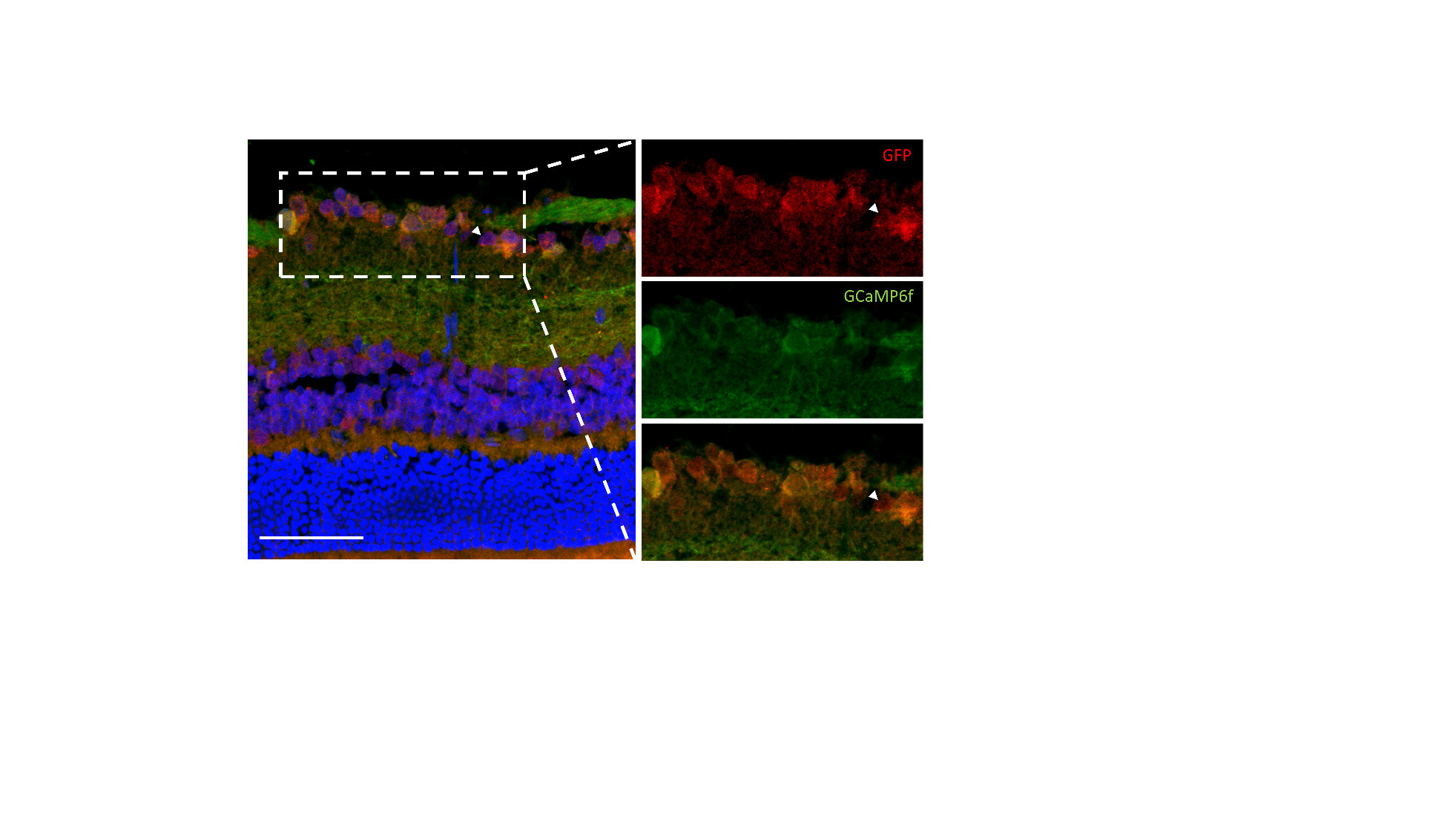


**Figure 3 Supp. Anti-GFP staining of GCaMP6f-LE retina**. Arrowheads mark an anti-GFP stained cell with no GCaMP6f fluorescence. Scale bar 50µm

**GCaMP6f-RCS rats exhibited a gradual loss of oscillatory potentials amplitude.**

Acquired ERG signals were bandpass filtered in the range of 80 to 160Hz to isolate oscillatory potentials (OP), which putatively originate from amacrine and ganglion cells and are an additional indicator of the retina’s functional viability. Representative OP signals induced by a 80Cds/cm^2^ are brought in Fig.4 Supp highlighting the decrease in the maximal amplitude throughout the degeneration process.


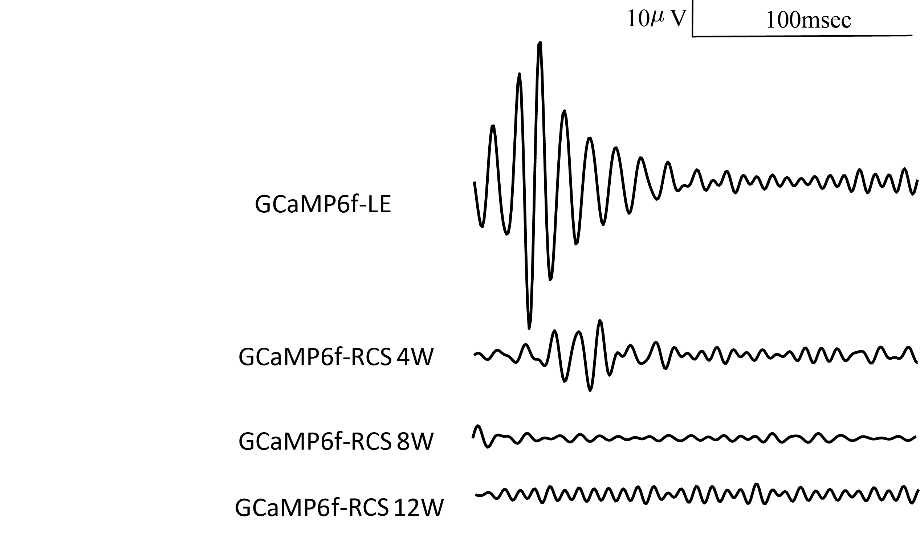


**Figure 4 Supp. Oscillatory potentials**. Representative oscillatory potentials for the various investigated animal groups, for a 80Cds/cm2 2msec flash.
